# Perceived Barriers to Healthy Eating and Physical Activity among Adolescents in Seven Arab Countries: A Cross-Cultural Study

**DOI:** 10.1155/2013/232164

**Published:** 2013-11-14

**Authors:** Abdulrahman O. Musaiger, Mariam Al-Mannai, Reema Tayyem, Osama Al-Lalla, Essa Y. A. Ali, Faiza Kalam, Mofida M. Benhamed, Sabri Saghir, Ismail Halahleh, Zahra Djoudi, Manel Chirane

**Affiliations:** ^1^Arab Centre for Nutrition, P.O. Box 26923, Manama, Bahrain; ^2^Department of Mathematics, College of Science, Sakhir, Bahrain; ^3^Department of Clinical Nutrition and Dietetics, Faculty of Allied Health Science, The Hashemite University, Zarqa, Jordan; ^4^Department of Nutrition and Health, Ministry of Education, Dubai, UAE; ^5^Elia Nutrition and Health Centre, Kuwait, Kuwait; ^6^Dietetic Clinic, Damascus, Syria; ^7^Department of Food Science, Faculty of Agriculture, University of Tripoli, Tripoli, Libya; ^8^Faculty of Agriculture, Hebron University, Hebron, Palestine; ^9^Nutrition Department, Makassed Hospital, Jerusalem, Palestine; ^10^NutriDar, Algiers, Algeria

## Abstract

*Objective.* To highlight the perceived personal, social, and environmental barriers to healthy eating and physical activity among Arab adolescents. *Method.* A multistage stratified sampling method was used to select 4698 students aged 15–18 years (2240 males and 2458 females) from public schools. Seven Arab counties were included in the study, namely, Algeria, Jordan, Kuwait, Libya, Palestine, Syria, and the United Arab Emirates. Self-reported questionnaire was used to list the barriers to healthy eating and physical activity facing these adolescents. *Results.* It was found that lack of information on healthy eating, lack of motivation to eat a healthy diet, and not having time to prepare or eat healthy food were the main barriers to healthy eating among both genders. For physical activity, the main barriers selected were lack of motivation to do physical activity, less support from teachers, and lack of time to do physical activity. In general, females faced more barriers to physical activity than males in all countries included. There were significant differences between males and females within each country and among countries for most barriers. *Conclusion.* Intervention programmes to combat obesity and other chronic noncommunicable diseases in the Arab world should include solutions to overcome the barriers to weight maintenance, particularly the sociocultural barriers to practising physical activity.

## 1. Introduction

Over the last decade, the unhealthy lifestyle and poor dietary habits have been of great concern to the local health authorities in most Arab countries. This is mainly due to the fact that these factors are among the leading causes of obesity and chronic noncommunicable diseases [1]. Statistics from the World Health Organization indicate that more than 60% of morbidity, disability, and mortality in these countries are caused by chronic noncommunicable diseases, especially cardiovascular disease, diabetes, and cancer [2]. Obesity has reached an epidemic rate in Arab countries for both children and adults. Among adolescents aged 15–18 years, the proportions that were overweight and obese in seven Arab countries ranged from 25% to 60% [3]. This current epidemic of childhood obesity is largely due to an environment that promotes excessive food consumption and encourages sedentary behaviours [4]. Therefore, weight maintenance such as promoting healthy eating and physical activity among adolescents contributes to improving the health status of children and most probably prevents obesity and many chronic diseases in adulthood [5].

Several studies in the Arab world have reported that the dietary habits of the people have become more westernized [6–8]. The diet of Arab adolescents is per se characterized by a low intake of fruit, vegetables, and milk and a high intake of sugar-sweetened beverages, fast foods, and sweets [9–11]. This type of diet is strongly associated with the incidence of obesity and other chronic diseases [1]. Among Syrian adolescents, for example, the percentage of the daily energy intake contributed by sweets and sugary beverages was significantly higher in obese than nonobese adolescents; whereas the energy intake from milk, other dairy products, and fruit was found to be significantly higher in nonobese than obese subjects [12]. The estimated daily intake of fruit and vegetables among young Arabs aged 15–29 years was reported to be 296 and 323 grams for males and females, respectively. Increasing the daily intake of fruit and vegetables to up to 600 grams, the baseline choice, could reduce the risk of some chronic diseases such as ischaemic heart disease, ischemic strokes, and some types of cancer [13]. Furthermore, more than 40% of Arab adolescents skipped breakfast [14]. It was evident that skipping breakfast is associated with high risk of obesity [15] and poor cardiometabolic health status [16]. 

Moreover, the lifestyle of Arab adolescents has changed to be more sedentary, with long durations spent on viewing television, playing video games, and using the internet, as well as lack of physical activity [6–8]. The majority of Arab adolescents do not meet the recommended guidelines for daily physical activity. It has been reported that more than 85% of girls and 75% of boys aged 13–15 years in seven Arab countries (Djibouti, Egypt, Jordan, Libya Morocco, Oman, and the United Arab Emirates) did not engage in a sufficient amount of daily physical activity (obtaining at least 60 minutes of physical activity per day) [17].

Giving the high rate of obesity among adolescents in the Arab world, in addition to an environment that encourages an unhealthy lifestyle and culture of eating, the need to study the barriers to a healthy lifestyle is essential. However, studies on obstacles to the adoption of healthy eating and a healthy lifestyle in Arab adolescents are extremely lacking. Data from western countries has indicated that there are various social, personal, cultural, and environmental barriers to healthy eating [18, 19] and physical activity [20, 21] among teens. Understanding barriers to a healthy lifestyle among adolescents is important in any intervention to promote the nutritional and health status of the community. Thus, the objective of this study is to provide a better understanding of the barriers to healthy eating and physical activity among adolescents in seven Arab countries.

## 2. Methods

### 2.1. Sampling and Participants

This study is part of the ARAB-EAT project, which aims to investigate prevalence of obesity, eating attitudes, and barriers to healthy eating and physical activity among adolescents in seven Arab countries: Algeria, Jordan, Kuwait, Libya, Palestine, Syria, and the United Arab Emirates (UAE). Students aged 15–18 years from public schools were the target group in this study. Data were obtained from one of the major cities in each country. The sample was calculated with a 5% margin of error and with 95% confidence intervals.

The students were selected using a multistage stratified sampling method. To ensure the representation of various social classes, each city was first divided into administrative regions, which varied from two to five regions, depending on the country. The schools were divided into boys and girls secondary schools, and only government schools were included. Private schools were excluded because of a lack of statistics on these schools in most of the included countries and difficulty in obtaining permission from some of the schools. The schools were then selected proportionally by a simple random method from each administrative region. The classes were selected for each secondary level (Levels 10 to 12) in each school using a simple random method. The total sample of students selected from each country varied, depending on the number of students in each class and number of selected schools.

To ensure the accuracy and consistency of the sampling procedure, the answer of the questionnaire, and the collection of data, a standardized protocol was prepared and distributed to all participating centres in the seven countries. Each centre was responsible for training its research team, as well as obtaining ethical approval from the government authorities concerned, mainly from the Ministries of Education. The total sample obtained from schools in the seven countries was 4698 (2240 males and 2458 females). Due to the difficulty in obtaining permission from one administrative region in Algiers, the capital of Algeria, the sample size represented two rather than three administrative regions in Algiers. This may have affected the findings concerning Algeria. Data were collected between March 2010 and January 2011.

### 2.2. Questionnaire

Statements relating to barriers to healthy eating and physical activity were obtained from a previously published study, which used a validated self-reported questionnaire on young women [22]. The statements were first translated into Arabic. Slight modifications were then carried out to adapt the statements to the Arabic culture as well as to the target group of this study (adolescents). For example, the words “partners” and “children” were deleted, and the words “parents” and “teachers” were added. For the environmental barriers to physical activity, four new statements were added to the original statements and one irrelevant statement was deleted. These four statements were related to the cultural, climate and economic barriers to physical activity in the Arab community. A statement which related to jobs was deleted from the section on the social and environmental barriers to healthy eating, as it was not applicable. The final version of the questionnaire consisted of 10 and 14 statements that were related to barriers to healthy eating and to physical activity, respectively. The barriers were divided into personal, social, and environmental barriers. Response options for all the barriers statements were *not a barrier*, a *somewhat important barrier*, and a *very important barrier*.

The Arabic version of the questionnaire was then tested on 628 Kuwaiti adolescents. The findings revealed that the questionnaire was well understood by the participants, and no significant modifications were made. To test the reliability of the questionnaire, Cronbach's alpha coefficient was calculated for each group of statements and compared with the original statements. It was found that Cronbach's alpha coefficients for the statements related to barriers to physical activity ranged from 0.711 to 0.762, while those related to barriers to healthy eating were between 0.652 and 0.686, indicating moderate internal reliability, which is very close to the original version (Table 1).

### 2.3. Data Analysis

Data were first entered in an Excel file and then sent to the central processing station (Bahrain), along with the questionnaire, for the data to be checked and the statistical analysis to be carried out. SPSS statistical package version 15 was used in the analysis of the data. Chi-square test was used to examine the association between males and females in healthy eating and physical activity barriers.

## 3. Results

Tables 2 and 3 present the proportions of adolescents reporting each of the perceived barriers to healthy eating in the seven Arab countries for males and females, respectively. With the exception of Palestinian males, “do not have enough information about a healthy diet” seems to be one of the main barriers among both males and females in all the countries included in the study. Combining the response categories of *somewhat important barrier* and *important barrier*, 67% to 75% of males and 64% to 82% of females reported this barrier. “Not having motivation to eat healthy diet” came next as a barrier to healthy eating among both males and females, except for males in Libya and Palestine. The proportion of those who reported this barrier as *somewhat important* or *important* ranged from 53% to 64% in males and 65% to 75% in females. Another barrier which was relatively highly reported was “do not have access to healthy foods,” where about 50% to 69% of males and 51% to 64% of females reported that this barrier was *somewhat important* or *important*.

As for social barriers to healthy eating, “not having time to prepare or eat healthy foods because of school commitment” was the most important barrier reported by both genders in all countries, with this being reported by a higher percentage of females than males. Algerian males (43%) and females (50.9%) were more likely to report this barrier as important, compared with their counterparts in other countries (the range was from 23.5% to 36.4% of males and from 32% to 40.4% in females in other countries). In general, there were significant differences between males and females in these seven countries (*P* < 0.05) for all barriers to healthy eating.

Perceived barriers to physical activity reported by male and female adolescents are presented in Tables 4 and 5, respectively. Interestingly, all the personal barriers were *not important* among males in Algeria, Jordan, Libya, Syria, and UAE, but they were *somewhat important* or *important* among males in Kuwait and Palestine. The picture was not the same for females, where about more than 50% of the females perceived the two barriers as *somewhat important *or *important* (“do not have motivation to do physical activity, exercise, or sport” and “Do not have skills to do physical activity, exercise, or sport”). However, “not enjoying physical activity, exercise, or sport” was perceived to be *not important* by 56%–74% of the females. Excluding males in Kuwait, the barrier “no parents' support to be physically active” was reported to be *not important* by 53%–70% of males and 44%–61% of females. The barrier of “not having the time to be physically active” was perceived to be *somewhat important* or *important* by the males in all countries, except in Jordan where the proportion was significantly lower (59% compared to more than 70% in other countries). However, this barrier seems to be more strongly perceived by all females (76%–89% reported that this barrier was *important* or *somewhat important*). In general, cultural factors were found to be *important* or *somewhat important* barriers among females but not among males. The differences between males and between females in the seven countries regarding the majority of barriers to physical activity were significant (*P* < 0.05).

The significance of differences between males and females in each country for perceived barriers to healthy eating and physical activity is presented in Tables 6 and 7, respectively. The difference between males and females concerning “do not have enough information about a healthy diet” was found to be highly statistically significant in Jordan, Kuwait, Libya, Palestine, and UAE (*P* values ranged from 0.006 to 0.000). However, there was great variation between males and females from country to country for the rest of the barriers. As for physical activity barriers, the differences between males and females were significant for most barriers. Almost all personal barriers to physical activity in all countries were highly significant for males and females, whereas the differences varied between countries with respect to social support barriers and environmental barriers.

## 4. Discussion

This study indicates that there are several personal, social, and environmental barriers to healthy eating and physical activity among adolescents in Arab countries, and there are significant differences in these barriers between males and females in each country and among countries. Lack of information on healthy eating, lack of motivation to eat healthy diets, and not having time to prepare or eat healthy foods due to school commitments were found to be the main barriers to healthy eating. However, lack of motivation to do physical activity, insufficient support from teachers, and lack of time to do physical activity were the main barriers to physical activity, especially among females. Parents and friends support for eating a healthy diet or to do physical activity were somewhat positive, while the support of teachers was indicated as negative, which suggests the role these people have in overcoming barriers to weight maintenance.

Many studies in the Arab world have reported that the dietary habits of adolescents are unhealthy and that it is therefore important to promote healthy eating as well as a healthy lifestyle [7–9]. However, based on the current study, a deficiency of information related to healthy nutrition was reported as being one of the predominant obstacles to eating a healthy diet. This barrier has also been stated by adolescents in western countries [23, 24]. This may indicate that there are insufficient nutrition education programmes, especially through the mass media and schools. Although studies on the effect of nutrition education programmes on food habits in the Arab world are scanty, some evidence has shown that these programmes have little impact on changing nutritional behaviours. This is probably due to the inadequate information provided, the lack of specialized people in nutrition education, and the inability of these programmes to attract the attention of the public [25]. Furthermore, it was found that much of the nutrition information in the school curricula in Arab countries is outdated and does not cover many local dietary habits that are associated with existing diet-related diseases in Arab states [26]. The Third Arab Conference on Nutrition, which was held in the United Arab Emirates in 2007, made several recommendations for promoting healthy nutrition in the Arab world. These recommendations included the need for the review and evaluation of the current curricula in both government and private schools in order to update the information related to nutrition and to link this information to the local and Arab situation [27].

Poor availability of healthy diets in schools and food outlets and preferences for fast foods and easy access to them may reduce the motivation of adolescents to eat healthy foods [5, 28]. The lack of personal motivation to eat a healthy diet has also been shown to be a barrier to adolescents in some western countries [29]. Motivation to practise healthy eating by school children is usually influenced by parents, peers, teachers, and the mass media [29, 30]. Food choices and availability at home are mostly influenced by parents [31]. The current study has shown that parents are not an important barrier to healthy eating; however, the lack of knowledge of sound nutrition among parents as well as their work schedule may continue to reduce the level of supervision and guidance of children's food habits [32]. At the adolescence stage, peers have a high impact on nutritional behaviour [33]; nevertheless, friends were not reported to be a barrier to healthy eating in this study. Shepherd et al. [5] reported that healthy food intake is mainly associated with parents and the home environment, while fast food intake is associated with friendship and socioeconomic status. Teachers seem to be an important barrier in countries included in this study. This finding is in agreement with other studies in western countries [20, 34]. Teachers who model unhealthy eating habits have been found to be a barrier to healthy eating [35]. Nutrition knowledge and the attitudes of teachers are of great concern in the promotion of healthy dietary habits among students [36]. Some studies have shown that school teachers lack nutrition information [37] and that they have an unhealthy lifestyle [38].

Physical inactivity, either among children or adults, is one of the fastest growing risk factors associated with several chronic diseases in the Arab states [1]. It is apparent from this study that Arab adolescents face more barriers to the practice of physical activity than they do to eating a healthy diet, and the differences between males and females for most physical activity barriers are highly significant. In general, personal and social barriers were found to be higher among females than males. Many barriers to physical activity among Arab adolescents have also been reported in western countries, and to a lesser extent among females in western countries than among females in Arab countries [21, 39, 40]. As with healthy eating barriers, support from parents, friends, and teachers for the practice of physical activity should be taken into consideration when tackling physical activity barriers. It has been shown that adolescents with little support from friends for physical activity and with physically inactive parents tend to be physically inactive [41], while adolescents who believe that their friends regard them as athletically competent have been found to exhibit a more positive feeling towards physical activity [42].

Several factors are associated with barriers to physical activity. Among US adolescents, Kahn et al. [43] found that the most important variables associated with the practice of physical activity in both genders were age, BMI, psychosocial factors, parental attitudes about physical activity, parental physical activity, parental attitudes towards body shape, perceived peer views of body shape, and environmental barriers. However, due to the sociocultural differences between western and Arab countries, not all these factors may be applicable to the Arab culture. Studies of factors associated with the practice of physical activity in the Arab world are very limited. The nutrition transition during the past decades in most Arab countries has led to a more sedentary lifestyle, especially with advances in technology and transportation. The appeal of television, electronic games, and computers has increased the sedentary time of children [6]. 

The highly significant differences between males and females in physical activity barriers in this study could be attributed to sociocultural factors. In general, women in most Arab states are facing more barriers to the practice of physical activity than men. There are greater freedom and more places for men to practise physical activity and other recreational activities than for women. Furthermore, due to religious and social norms, most women in the Arab region cannot practise exercise outdoors and with sports dress, as many families do not allow their girls to practise exercise outdoors for religious and safety reasons. Also, many families do not permit their girls to practise physical activity wearing sports dress, but with traditional dress, which is not physically comfortable for taking exercise; this in turn discourages them from exercising outdoors [44]. In Bahrain, it was reported that 67% of women believed that there is sex discrimination in the lack of opportunities for women to take part in physical activity, as most exercise and sports facilities are provided for men. Approximately 24% of these women perceived that the negative attitudes of the community and family members towards women who practise exercise are preventing them from exercising [45].

The variation in barriers to healthy eating and to physical activity among adolescents in the seven Arab countries may be due to the differences in socioeconomic and cultural factors, as well as the prevalence of obesity in these countries. Some countries with high economic status such as Kuwait and UAE have experienced nutrition transition earlier than other countries, which leads to rapid change in dietary habits and lifestyle. Consequently, the prevalence of obesity and sedentary behaviours is relatively high, which in turn affects the barriers to a healthy lifestyle in adolescents. However, the sociocultural differences should be considered, as these factors are not equal in these countries. This is beyond the scope of this paper. 

This study has some limitations that are worth mentioning. First, the sample did not include private schools, which means that the sample did not represent all secondary school students in the studied Arab countries. Second, the questionnaire should have asked for more information on sociocultural barriers, especially those related to physical activity. Third, the questionnaire may need further validation. Despite these limitations, this study is the first attempt to investigate barriers to healthy eating and physical activity in a relatively large sample of adolescents in various Arab countries and provide baseline data for any further studies of this aspect. 

In conclusion, the current environment in Arab countries is characterized by the high availability of unhealthy foods coupled with a lifestyle requiring a low level of physical activity. This environment is promoting a high energy intake and low energy expenditure. Therefore, to combat the epidemic of obesity and other chronic diseases, we must first correct the environment. The results of this study highlighted some of the barriers that are associated with negative nutrition and unhealthy lifestyles in Arab adolescents. A strategy to overcome barriers to healthy eating and physical activity in Arab schoolchildren should take into consideration the support from parents, peers, and teachers, time management, self-motivation, increased nutrition awareness, sociocultural variables, and the provision of facilities for adolescents to practise physical activity in and out schools. Increasing the availability of healthy foods in school canteens and providing an environment that encourages physical activity are also essential elements for supporting a healthy lifestyle among adolescents. We hope that this study opens the door for further in-depth studies related to factors that inhibit people in the Arab world from eating healthy foods and taking part in physical activity.

## Figures and Tables

**Table 1:** Reliability of Arabic version of questionnaire as compared with original English version, using Cronbach's alpha coefficients.

Barriers statements	Cronbach's alpha coefficients	
	Arabic version	English version
Barriers to physical activity		
Personal barriers (3 statements)	0.762	0.760
Social support barriers (3 statements)	0.711	0.680
Environmental barriers (8 statements)	0.720	0.710
Barriers to healthy eating		
Personal barriers (6 statements)	0.686	0.830
Social and environmental barriers (4 statements)	0.652	0.720

**Table 2:** Perceived barriers to healthy eating among adolescents of seven Arab countries (male) (%).

Barriers	Algeria			Jordan			Kuwait			Libya			Palestine			Syria			UAE		
	Imp.	Som.	Not.	Imp.	Som.	Not.	Imp.	Som.	Not.	Imp.	Som.	Not.	Imp.	Som.	Not.	Imp.	Som.	Not.	Imp.	Som.	Not.
(A) Personal and environmental barriers to healthy eating																					
(1) Do not have enough information about a healthy diet	25.6	40.5	33.8	26.7	44.8	30.5	32.5	41.9	25.6	22.9	49.6	27.5	30.7	21.6	47.7	27.2	46.5	26.4	25.6	49.9	25.5
(2) Do not have motivation to eat a healthy diet	27.7	25.6	46.7	21.0	33.5	45.5	22.7	40.9	36.4	16.1	33.2	50.7	25.5	23.5	51.0	20.5	37.2	42.3	19.2	40.2	40.6
(3) Do not enjoy eating healthy foods	22.1	35.4	42.6	21.7	33.7	44.6	21.7	46.2	32.2	17.5	33.6	48.7	29.4	31.4	39.2	16.5	38.0	45.5	24.4	39.1	36.5
(4) Do not have skills to plan and shop for preparing or cooking healthy foods	36.4	31.3	32.3	34.1	36.9	29.0	31.1	42.7	26.2	38.9	33.9	27.2	27.5	23.5	49.0	40.4	36.6	22.9	31.2	37.6	31.2
(5) Do not have access to healthy foods	36.9	19.5	43.6	23.8	31.1	45.1	25.8	44.8	29.4	25.4	24.3	50.3	35.3	33.3	31.4	24.9	35.0	40.0	22.2	35.7	42.1
(6) Not able to buy healthy foods that are inexpensive	20.5	20.5	59.0	10.3	22.7	67.0	18.9	32.9	48.3	11.4	20.0	68.6	27.5	21.6	50.9	15.1	29.6	55.3	9.8	20.7	69.5

(B) Social barriers to healthy eating																					
(1) No parents' support to eat a healthy diet	11.3	15.4	73.3	6.9	21.2	71.9	17.5	37.1	45.5	05.7	16.4	77.9	15.7	19.6	64.7	07.6	23.1	69.2	12.0	21.1	66.9
(2) No friends' support to eat a healthy diet	32.3	26.7	41.0	18.5	30.0	51.5	21.7	37.1	41.2	22.5	30.0	47.5	19.6	17.6	62.8	20.5	34.8	44.7	19.2	39.1	41.7
(3) No teachers' support to eat a healthy diet	29.2	25.1	45.6	23.8	27.0	49.2	32.5	40.2	29.3	33.6	22.5	43.9	31.4	05.9	62.7	21.9	29.4	48.7	20.3	30.5	49.2
(4) Not having time to prepare or eat healthy foods because of school commitment	43.1	32.3	24.6	23.6	30.9	45.5	36.4	35.0	28.7	31.8	33.6	34.6	23.5	41.2	35.3	33.2	37.8	29.0	31.6	30.8	37.6

Imp.: important barrier (%), Som.: somewhat important (%), and Not.: not a barrier (%).

**Table 3:** Perceived barriers to healthy eating among adolescents of seven Arab countries (female) (%).

Barriers	Algeria			Jordan			Kuwait			Libya			Palestine			Syria			UAE		
	Imp.	Som.	Not.	Imp.	Som.	Not.	Imp.	Som.	Not.	Imp.	Som.	Not.	Imp.	Som.	Not.	Imp.	Som.	Not.	Imp.	Som.	Not.
(A) Personal and environmental barriers to healthy eating																					
(1) Do not have enough information about a healthy diet	34.3	37.0	28.7	35.8	45.8	18.4	21.4	47.5	31.2	42.9	37.7	19.4	30.0	34.0	36.0	33.0	43.7	22.4	14.0	55.9	30.1
(2) Do not have motivation to eat a healthy diet	23.0	23.4	53.6	21.6	38.0	40.4	24.2	38.8	40.0	22.0	31.1	46.9	31.0	30.0	39.0	20.3	36.3	43.5	17.5	51.1	31.4
(3) Do not enjoy eating healthy foods	29.8	26.8	43.4	23.9	35.8	40.4	27.8	37.9	34.6	20.3	35.4	41.7	31.0	19.0	50.0	23.2	37.8	39.0	17.5	45.5	37.0
(4) Do not have skills to plan and shop for preparing or cooking healthy foods	37.4	34.0	28.7	38.7	34.9	26.4	31.2	36.2	32.6	30.0	35.2	34.8	31.0	34.0	35.0	38.6	35.9	25.5	25.2	43.7	31.1
(5) Do not have access to healthy foods	38.5	22.3	39.2	26.2	33.9	39.9	22.2	31.8	46.0	34.6	22.3	43.1	38.0	26.0	36.0	29.2	26.9	43.9	19.2	31.8	49.0
(6) Not able to buy healthy foods that are inexpensive	11.7	22.3	66.0	5.6	18.4	75.9	5.1	18.5	76.4	07.7	20.9	71.4	21.0	22.0	57.0	10.7	25.3	63.9	4.9	15.0	80.1

(B) Social barriers to healthy eating																					
(1) No parents' support to eat a healthy diet	08.3	10.2	81.5	9.8	20.5	69.7	10.7	23.3	66.0	12.6	19.1	68.3	12.0	14.0	74.0	10.9	16.8	72.3	8.7	22.4	68.9
(2) No friends' support to eat a healthy diet	32.1	26.4	41.5	17.6	36.2	46.2	19.7	23.3	57.0	32.0	28.0	40.0	16.0	26.0	59.0	22.8	30.2	47.0	19.2	37.8	43.0
(3) No teachers' support to eat a healthy diet	26.0	23.6	50.2	19.3	30.3	50.4	20.5	30.6	48.9	31.4	20.9	47.7	27.0	13.0	60.0	17.9	27.1	55.0	14.3	32.2	53.5
(4) Not having time to prepare or eat healthy foods because of school commitment	50.9	31.3	17.7	32.0	38.1	29.9	40.4	32.6	27.0	37.1	36.3	26.6	48.0	35.0	17.0	41.3	36.3	22.4	36.0	37.8	26.2

Imp.: important barrier (%), Som.: somewhat important (%), and Not.: not a barrier (%).

**Table 4:** Perceived barriers to physical activity among adolescents of seven Arab countries (male) (%).

Barriers	Algeria			Jordan			Kuwait			Libya			Palestine			Syria			UAE		
	Imp.	Som.	Not.	Imp.	Som.	Not.	Imp.	Som.	Not.	Imp.	Som.	Not.	Imp.	Som.	Not.	Imp.	Som.	Not.	Imp.	Som.	Not.
(A) Personal barriers to physical activity																					
(1) Do not have motivation to do physical activity, exercise, or sport	7.7	12.3	80.0	7.1	14.6	78.3	20.3	34.4	45.5	04.3	14.3	85.0	17.6	19.6	62.7	6.0	18.7	75.3	5.6	22.9	71.4
(2) Not enjoying physical activity, exercise, or sport	5.1	07.7	87.2	3.9	12.0	84.1	15.7	35.0	49.3	05.0	08.9	86.1	27.5	17.6	54.9	7.4	09.1	83.3	4.1	17.3	78.6
(3) Do not have the skills to do physical activity, exercise, or sport	6.2	13.8	80.0	8.4	24.5	67.1	22.4	35.7	41.9	10.7	17.9	71.4	29.4	29.4	41.2	9.3	25.4	65.4	10.9	29.7	59.4

(B) Social support barriers to physical activity																					
(1) No parents' support to be physically active	8.7	21.0	70.3	15.2	23.6	61.2	23.8	35.7	40.5	12.9	27.5	59.6	29.4	17.6	53.0	15.3	30.8	53.9	15.8	24.8	59.4
(2) No friends' support to be physically active	9.2	21.0	69.7	14.6	26.8	58.6	19.2	34.3	46.5	11.8	26.4	61.8	23.5	27.5	49.0	15.7	25.2	59.2	10.9	24.8	64.3
(3) No teachers' support to be physically active	30.3	23.6	46.2	32.6	27.0	40.4	29.0	34.6	36.4	39.3	23.2	37.5	35.3	15.7	49.0	30.2	31.8	38.0	15.8	26.7	57.5

(C) Environmental barriers to physical activity																					
(1) Do not have enough information about how to increase physical activity	16.9	30.3	52.8	20.0	33.0	47.0	32.2	38.5	29.8	15.0	37.5	47.5	29.4	19.6	51.0	23.7	38.2	38.0	18.2	41.0	40.6
(2) Not having access to places to do physical activity, exercise, and sport	32.8	17.9	49.2	20.4	29.0	50.6	26.6	34.6	38.8	23.6	22.1	54.3	39.2	19.6	58.8	22.7	29.4	47.9	13.5	25.9	60.5
(3) Not being able to find physical activity facilities that are inexpensive	24.6	27.7	47.7	19.5	30.9	49.6	26.9	35.3	37.8	23.6	28.9	47.5	33.3	27.5	39.2	23.9	31.8	44.3	14.3	29.7	56.0
(4) Not having the time to be physically active	45.1	32.3	22.6	21.7	37.6	40.8	37.1	40.6	22.3	36.8	36.1	27.1	49.0	27.5	23.5	29.4	45.3	25.4	39.0	36.1	24.8
(5) Feeling shy when practising exercise outdoors	05.6	04.6	89.7	5.5	12.7	81.8	19.2	35.0	45.8	06.4	12.5	81.1	13.7	25.5	60.8	07.6	14.7	77.7	06.8	21.1	72.1
(6) The climate is not suitable for practising exercise	05.1	32.3	62.6	6.7	41.9	51.5	41.6	40.2	18.2	02.9	46.1	51.0	21.6	39.2	39.2	08.0	38.8	53.1	18.4	53.4	28.2
(7) Not being able to practise physical activity due to cultural factors	04.6	02.6	92.8	8.2	12.0	79.8	16.0	21.3	65.7	05.4	07.5	87.1	21.6	15.7	62.7	10.7	16.1	73.2	07.1	09.8	83.1
(8) Do not have enough money to enrol on physical activity club	10.8	17.4	71.8	8.8	18.5	72.7	23.4	29.4	47.2	10.7	11.8	77.5	33.3	13.7	51.0	14.9	24.7	60.4	11.3	14.3	74.4

Imp.: important barrier (%), Som.: somewhat important (%), and Not.: not a barrier (%).

**Table 5:** Perceived barriers to physical activity among adolescents of seven Arab countries (female) (%).

Barriers	Algeria			Jordan			Kuwait			Libya			Palestine			Syria			UAE		
	Imp.	Som.	Not.	Imp.	Som.	Not.	Imp.	Som.	Not.	Imp.	Som.	Not.	Imp.	Som.	Not.	Imp.	Som.	Not.	Imp.	Som.	Not.
(A) Personal barriers to physical activity																					
(1) Do not have motivation to do physical activity, exercise, or sport	16.2	27.2	56.6	7.7	42.5	49.8	14.3	42.7	43.0	16.3	31.4	52.2	19.0	35.0	46.0	13.5	41.3	45.2	12.6	47.6	39.8
(2) Not enjoying physical activity, exercise, or sport	07.9	17.7	74.3	10.1	25.1	64.8	12.6	27.8	59.5	08.9	25.4	65.7	10.0	23.0	67.0	13.3	23.8	63.0	11.5	32.9	55.6
(3) Do not have the skills to do physical activity, exercise, or sport	19.6	32.1	48.3	18.8	37.7	43.5	20.2	36.8	43.0	23.7	37.4	38.8	24.0	27.0	49.0	28.3	39.4	32.4	18.2	50.3	29.7

(B) Social support barriers to physical activity																					
(1) No parents' support to be physically active	22.6	16.2	61.1	17.2	27.4	55.4	17.7	29.2	53.1	26.6	25.1	48.3	26.0	25.0	49.0	24.4	26.5	49.1	22.4	33.6	44.1
(2) No friends' support to be physically active	29.1	23.8	47.2	25.7	33.7	40.6	21.9	37.4	40.7	40.0	22.9	62.9	23.0	29.0	48.0	33.3	29.0	37.6	29.0	36.0	35.0
(3) No teachers' support to be physically active	30.2	22.6	47.2	34.9	30.8	34.3	25.8	36.2	38.0	36.3	17.4	46.3	28.0	16.0	56.0	34.9	27.7	37.4	21.0	37.8	41.3

(C) Environmental barriers to physical activity																					
(1) Do not have enough information about how to increase physical activity	32.1	33.6	34.3	32.8	39.7	27.5	26.7	41.0	32.3	34.9	37.1	28.0	31.0	36.0	33.0	36.8	28.8	24.4	26.9	52.1	21.0
(2) Not having access to places to do physical activity, exercise, and sport	47.9	16.6	35.5	26.2	30.5	43.3	29.8	33.4	36.8	34.6	19.4	54.0	38.0	23.0	39.0	32.7	26.3	40.9	26.2	35.7	38.1
(3) Not being able to find physical activity facilities that are inexpensive	36.6	23.0	40.4	18.2	34.3	47.5	21.3	31.7	46.9	24.6	23.1	52.3	44.0	23.0	33.0	25.5	29.2	45.2	21.7	30.8	42.6
(4) Not having the time to be physically active	67.9	21.5	10.6	38.5	37.9	23.6	52.5	30.9	16.6	47.7	31.7	20.6	64.0	21.0	15.0	48.5	30.6	20.9	50.3	30.4	19.2
(5) Feeling shy when practising exercise outdoor	15.1	23.4	61.5	11.9	17.0	71.1	18.5	25.8	52.3	21.7	23.4	54.9	27.0	23.0	49.0	17.2	23.6	59.3	17.8	33.9	48.3
(6) The climate is not suitable for practising exercise	14.7	41.1	44.2	11.3	37.9	50.8	43.3	36.0	20.7	10.9	42.6	46.5	24.0	43.0	33.0	14.4	40.7	45.0	50.7	30.1	19.2
(7) Not being able to practise physical activity due to cultural factors	29.1	11.3	59.6	19.9	26.6	53.5	28.4	23.0	48.6	52.0	21.1	26.9	65.0	16.0	19.0	35.9	19.1	45.0	37.4	19.6	43.0
(8) Do not have enough money to enrol on physical activity club	15.5	17.4	67.2	8.1	18.6	73.3	10.4	21.3	68.3	13.1	16.0	70.9	29.0	23.0	48.0	15.6	23.4	61.0	08.7	22.0	69.3

Imp.: important barrier (%), Som.: somewhat important (%), and Not.: not a barrier (%).

**Table 6:** Significant differences (*P* value) between males and females for perceived barriers to physical activity among Arab adolescents (using chi-square test).

Barriers	Algeria	Jordan	Kuwait	Libya	Palestine	Syria	UAE
(A) Personal barriers to physical activity							
(1) Do not have motivation to do physical activity, exercise, or sport	0.000	0.000	0.049	0.000	0.000	0.000	0.000
(2) Not enjoying physical activity, exercise, or sport	0.002	0.000	0.040	0.000	0.000	0.000	0.000
(3) Do not have the skills to do physical activity, exercise, or sport	0.000	0.000	0.799	0.000	0.000	0.000	0.000

(B) Social support barriers to physical activity							
(1) No parents' support to be physically active	0.000	0.188	0.008	0.000	0.148	0.001	0.002
(2) No friends' support to be physically active	0.000	0.000	0.317	0.000	0.930	0.000	0.000
(3) No teacher' support to be physically active	0.966	0.147	0.671	0.054	0.206	0.187	0.001

(C) Environmental barriers to physical activity							
(1) Do not have enough information about how to increase physical activity	0.000	0.000	0.328	0.000	0.000	0.000	0.000
(2) Not having access to places to do physical activity, exercise, or sport	0.003	0.048	0.699	0.090	0.653	0.002	0.000
(3) Not being able to find physical activity facilities that are inexpensive	0.024	0.539	0.071	0.247	0.055	0.782	0.014
(4) Not having the time to be physically active	0.000	0.000	0.001	0.018	0.004	0.000	0.037
(5) Feeling shy when practising exercise outdoor	0.000	0.000	0.060	0.000	0.001	0.000	0.000
(6) The climate is not suitable for practising exercise	0.000	0.037	0.054	0.000	0.377	0.002	0.000
(7) Not being able to practise physical activity due to cultural factors	0.000	0.000	0.000	0.000	0.000	0.000	0.000
(8) Do not have enough money to enrol on physical activity club	0.334	0.895	0.000	0.162	0.043	0.782	0.059

**Table 7:** Significant differences (*P* value) between males and females for perceived barriers to healthy eating among Arab adolescents (using chi-square test).

Barriers	Algeria	Jordan	Kuwait	Libya	Palestine	Syria	UAE
(A) Personal and environmental barriers to healthy eating							
(1) Do not have enough information about a healthy diet	0.128	0.000	0.006	0.000	0.007	0.055	0.006
(2) Do not have motivation to eat healthy diet	0.321	0.248	0.688	0.173	0.031	0.924	0.040
(3) Do not enjoy eating healthy foods	0.71	0.406	0.077	0.310	0.005	0.002	0.131
(4) Do not have skills to plan and shop for preparing and cooking healthy foods	0.683	0.331	0.155	0.036	0.006	0.619	0.251
(5) Do not have access to healthy foods	0.607	0.298	0.000	0.042	0.214	0.019	0.289
(6) Not able to buy healthy foods that are inexpensive	0.035	0.004	0.000	0.282	0.214	0.014	0.017

(B) Social barriers to healthy eating							
(1) No parents' support to eat healthy diet	0.108	0.263	0.000	0.006	0.084	0.015	0.496
(2) No friends' support to eat healthy diet	0.995	0.125	0.000	0.026	0.090	0.281	0.946
(3) No teachers' support to eat healthy diet	0.611	0.215	0.000	0.638	0.032	0.110	0.223
(4) Not having time to prepare or eat healthy foods because of school commitment	0.131	0.000	0.589	0.083	0.000	0.012	0.022
